# Synthetic Tactile Sensor for Macroscopic Roughness Estimation Based on Spatial-Coding Contact Processing

**DOI:** 10.3390/s25082598

**Published:** 2025-04-20

**Authors:** Muhammad Irwan Yanwari, Shogo Okamoto

**Affiliations:** 1 Department of Computer Science, Tokyo Metropolitan University, Hino 1910065, Japan; yanwari-muhammad@ed.tmu.ac.jp; 2 Department of Electrical Engineering, Politeknik Negeri Semarang, Kota Semarang 50275, Indonesia

**Keywords:** spatial frequency, macroscopic surface texture, PLS regression

## Abstract

Traditional tactile sensors primarily measure macroscopic surface features but do not directly estimate how humans perceive such surface roughness. Sensors that mimic human tactile processing could bridge this gap. This study proposes a method for predicting macroscopic roughness perception based on a sensing principle that closely resembles human tactile information processing. Humans are believed to assess macroscopic roughness based on the spatial distribution of subcutaneous deformation and resultant neural activities when touching a textured surface. To replicate this spatial-coding mechanism, we captured distributed contact information using a camera through a flexible, transparent material with fingerprint-like surface structures, simulating finger skin. Images were recorded under varying contact forces ranging from 1 N to 3 N. The spatial frequency components in the range of 0.1–1.0 mm^−1^ were extracted from these contact images, and a linear combination of these components was used to approximate human roughness perception recorded via the magnitude estimation method. The results indicate that for roughness specimens with rectangular or circular protrusions of surface wavelengths between 2 and 5 mm, the estimated roughness values achieved an average error comparable to the standard deviation of participants’ roughness ratings. These findings demonstrate the potential of macroscopic roughness estimation based on human-like tactile information processing and highlight the viability of vision-based sensing in replicating human roughness perception.

## 1. Introduction

The influence of texture perception on consumer purchase decisions and product evaluation is well documented. Tactile impressions can enhance or diminish a product’s perceived value or preference [1,2,3,4,5]. To automate the design or inspection process, a sensory system capable of emulating human perception is essential.

Thus far, many measurement systems have been proposed to estimate tactile sensations perceived by humans [1,6,7,8,9,10,11]. For instance, Richardson et al. [6] utilized a commercial multi-modal tactile sensor to predict the probability distributions of subjective scores describing the tactile properties of materials. Their system established a correspondence between the tactile sensor outputs and human perceptual ratings. Similarly, Hashim et al. [10] estimated perceived material properties, such as surface roughness, hardness, and thermal characteristics by integrating outputs from multiple measurement systems, including friction measurement devices, indentation testers, and profilometers. Among these studies, fabric hand value has the longest research history and links the mechanical and thermal properties of fabrics with subjective tactile sensations [1,12].

In this study, we focus on the perception of surface roughness, one of prominent tactile modalities [13,14]. When estimating perceived roughness using sensors, many tactile sensors utilize vibrational information generated as the sensor probe moves across the surface [9,10,11,15,16,17,18,19,20,21,22,23,24]. This approach aligns with the principle of temporal coding [25,26], wherein humans perceive microscopic roughness by relying on skin vibrations induced when a finger slides over a textured surface. In this context, these tactile sensors employ a roughness evaluation method that corresponds to human perceptual mechanisms for microscopic roughness, which refers to surface roughness features with asperity spacing on the order of several hundred micrometers or less.

On the other hand, in macroscopic roughness perception, the principle of spatial coding [25] is dominant. Macroscopic roughness refers to surface roughness where the spacing between protrusions exceeds several hundred micrometers. When the fingertip contacts such rough surfaces, the spatial distribution of skin deformation is detected by slowly adapting type I (SAI) mechanoreceptive units, which respond to static pressure or strain energy [27,28,29]. These units are densely distributed in the epidermis layer beneath the fingertip, with several dozen units present within an area of approximately 1 cm^2^ [30,31]. The spatial distribution of their activity correlates with the perceived intensity of macroscopic roughness [32,33,34]. Since the deformation distribution within the subcutaneous tissue is closely related to the deformation distribution on the skin surface, it is possible to estimate the perceived intensity of macroscopic roughness based on the spatial frequency spectrum of skin surface deformation [35,36]. A point of this roughness coding is that the human finger does not need to slide over textured surfaces; however, the pressing motion is satisfactory to capture the macroscopic surface roughness [37,38,39]. In other words, roughness information that does not require a sliding motion for perception is regarded as macroscopic roughness.

While the estimation of perceived intensity for microscopic roughness based on the principle of temporal coding has been widely implemented in engineering field, there are few examples of estimating macroscopic roughness perception using spatial coding, while a simulation study was reported previously [40]. Although previous studies have associated roughness perception with surface topography images, which contain spatial distribution information of surface protrusions [3,41], these approaches did not adhere to the spatial roughness coding mechanism. For example, Chen et al. [3] calculated average surface roughness values from topography images of textures to explain perceived roughness.

This study aims to estimate macroscopic roughness perceived by humans using a tactile sensor based on the principle of spatial coding. As mentioned earlier, tactile sensors employing this principle are extremely rare. To achieve this, it is necessary to acquire distributed deformation information from a sensor probe that mimics the structure and stiffness of a human fingertip in contact with rough surfaces. For this purpose, an image sensor (camera) is the most practical option. The method of capturing soft material deformations using an image sensor has been widely employed in tactile sensing [42,43,44,45,46]. However, most existing approaches focus on accurately measuring the topography of the contact surface rather than estimating roughness perception.

In this study, a transparent resin pad with stiffness similar to that of a human finger was pressed against surfaces with macroscopic roughness. We then computed the spatial frequency components of the contact regions from camera images. By applying a weighted summation to these computed frequency components, we estimated macroscopic roughness as perceived by humans. The weights were determined through statistical learning to ensure that the frequency components accurately reflect perceived roughness intensity, as measured in a separate experiment involving human participants.

The pad used in our study is equipped with fingerprint-like patterns similar to those of a human finger. Although these patterns are not necessary for estimating macroscopic roughness, they are useful for estimating microscopic roughness during sliding motions. To allow for future extension of the sensor toward broader-band surface roughness estimation, we deliberately included fingerprint structures on the pad (see Section 2.2).

In the field of neurophysiology, discussions often center on the relationship between individual spatial frequency components of neural activities of SAI units or skin deformation and human roughness perception [32,33,34,36]. However, no consensus has been reached regarding the most effective frequency components across different studies. Given this context, an approach that links multiple frequency components to roughness perception is more rational.

This approach may represent the most fundamental method for estimating the intensity of perceived macroscopic roughness. Nevertheless, its application in tactile sensors has been limited. By exploring the engineering applications of spatial coding for roughness estimation, this study is significant in advancing the field. Furthermore, clarifying its capabilities provides valuable insights for the broader tactile measurement community.

## 2. Apparatus

### 2.1. Workbench Configuration

**Figure 1:**
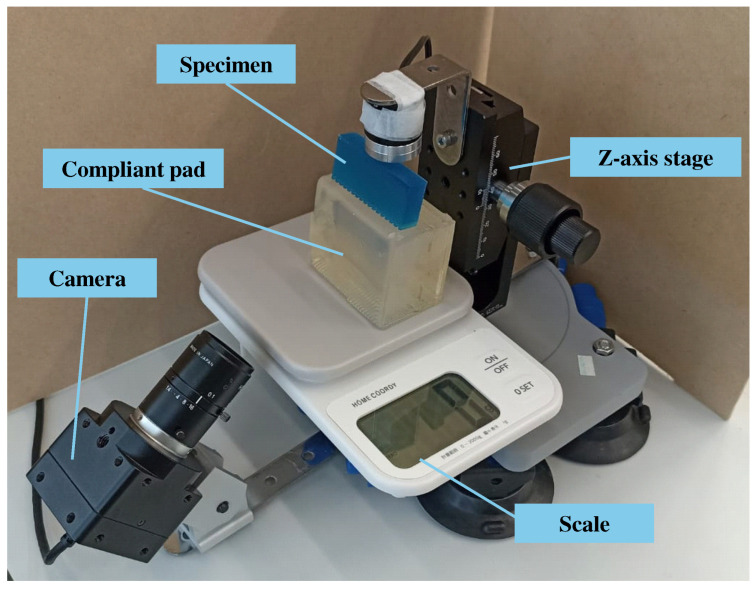
Workbench configuration. The camera was positioned to capture images of the contact area on the compliant plastic pad. A *z*-axis stage applied pressing force vertically from above the specimen.

The workbench setup is shown in Figure 1. A camera (L-836, Hozan Co., Ltd., Osaka, Japan; 1920 × 1080 pixels) was mounted on a metal frame at a 45-degree angle, positioned to capture the contact surface between the roughness specimen and the compliant pad, the details of which are described in Section 2.2. The contact area was captured from the side of the transparent pad. The camera was angled to reduce the amount of scattered light. The camera was fitted with a lens (L-600, Hozan Co., Ltd., Japan), capturing an image area of 5.5 cm × 3.1 cm, corresponding to a resolution of 349 pixels per centimeter.

To apply pressing force to the pad and specimen, a *z*-axis stage (LV-912S, MISUMI Co., Ltd., Tokyo, Japan) was utilized. A digital scale (Home Coordy 2 kg, AEON Topvalu Co., Ltd., Chiba, Japan) was employed to measure the applied force. The scale enabled the measurement of the force at the moment each image was captured.

### 2.2. Transparent and Compliant Pad with Ridges

**Figure 2:**
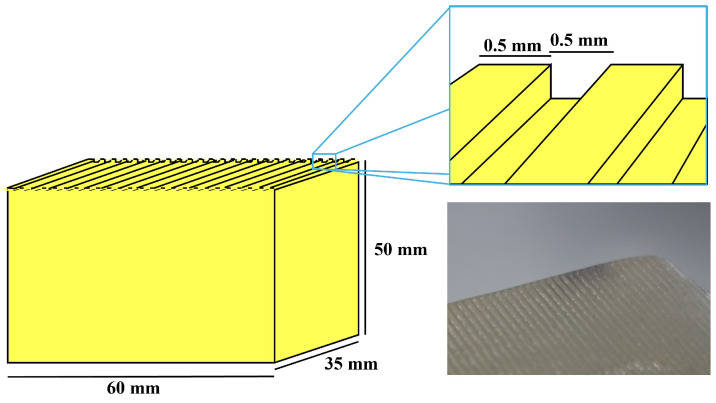
Transparent compliant block with fingerprint-like ridges, made from polyvinyl chloride plastisol.

A transparent compliant block with ridges was designed, as shown in Figure 2. The block had a box-like shape with dimensions of 60 mm in length, 35 mm in width, and 50 mm in height. Its surface featured a fingerprint-like pattern, with ridge and groove widths of 0.5 mm. These dimensions were approximately twice the size of human adult fingerprints [47]. Further downsizing is anticipated in future iterations.

The block was fabricated using polyvinyl chloride plastisol (Plastic Worm, Two-L Co., Ltd., Shiogama, Japan), a material with a Young’s modulus of 0.17 MPa, which closely resembles the softness of the fat layer in the human finger [48]. The material was cast into a female mold produced via additive manufacturing using a Form 3 printer (Formlabs Inc., Somerville, MA, USA).

Notably, the human finger consists of multiple layers with varying stiffness. Some previous studies have mimicked this structure by employing a two-layer configuration [18,24,49]. However, for the purpose of this proof-of-concept study, our pad adopts a simplified single-layer structure. Accordingly, the potential effects of this simplification on roughness estimation are not addressed in the present work.

The integration of fingerprint-like features into tactile sensors has been a focus of research for decades [50,51]. Finger ridges play a crucial role in active tactile exploration, such as sliding, by amplifying signals from surface microscopic asperities [24,52,53,54,55,56,57]. During grasping, these ridges enhance grip stability and contribute to even force distribution [54,58,59,60]. Notably, fingerprints are not required for estimating macroscopic surface features. In fact, they may introduce noise in vision-based sensing methods—particularly when their dimensions exceed those of actual human fingerprints. Specifically, the 1 mm period synthetic fingerprints used in this study can interfere with accurate imaging of surface roughness that includes spatial patterns near 1 mm in scale. Nevertheless, to explore the possibility of integrating sensing capabilities for both macroscopic (image-based) and microscopic (vibration-based) roughness estimation, the compliant pad in this study was intentionally equipped with fingerprint-like patterns.

To clarify the effect of synthetic fingerprints on image-based roughness sensing, we conducted a comparative test using a smooth pad made of the same plastic material. The smooth pad yielded clearer contact images, which may contribute to improved estimation accuracy.

### 2.3. Roughness Specimens

**Figure 3:**
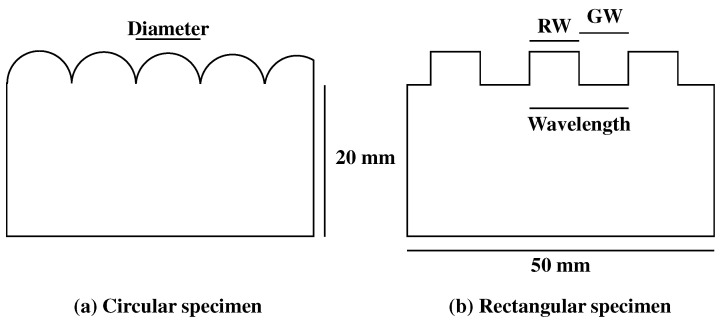
Dimensions of the specimens. Circular specimens are defined by the diameter of the semi-cylindrical gratings, while rectangular specimens are defined by the wavelength (the combined width of a ridge and groove). The ridge and groove widths are identical.

**Figure 4:**
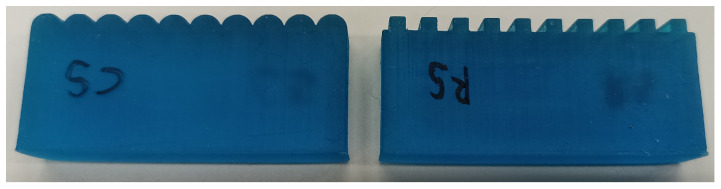
Examples of circular and rectangular specimens. (**Left**) Circular grating with a diameter of 5 mm. (**Right**) Rectangular grating with ridge and groove widths of 2.5 mm.

The roughness specimens used in this experiment were fabricated via additive manufacturing (Form 3, Formlabs Inc., Somerville, MA, USA) using Tough Resin V5 (Formlabs Inc., Somerville, USA). Each specimen measured 50 mm in length, 20 mm in height, and 10 mm in width, as shown in Figure 3 and Figure 4. To enhance the study’s generalizability, two types of roughness features were employed: circular and rectangular.

For the circular gratings, seven diameter levels were designed, ranging from 2.0 mm to 5.0 mm in increments of 0.5 mm. Unlike typical dotted roughness scales [32,33], the hemi-circular bumps were arranged without spacing and were characterized by a single parameter: diameter. Similarly, the rectangular specimens featured seven surface wavelength levels, also ranging from 2.0 mm to 5.0 mm in 0.5 mm increments. The rectangular specimens had a 1:1 ratio between ridge width (RW) and groove width (GW). Researchers largely agree that macroscopic roughness consists of surface features with spatial periods ranging from few hundred micrometers to 1.0 mm or greater [25,34,61,62]. Hence, specimens with surface wavelengths below 2.0 mm should have been covered in this study. However, the fingerprint patterns on the compliant plastic pad (with a period of 1.0 mm) were approximately twice the size of actual human fingerprints. These synthetic fingerprints were clearly visible in the camera images and interfered with features exhibiting spatial periods of approximately 1.0 mm. Therefore, we set the minimum roughness period to 2.0 mm. This limitation should be addressed by downsizing the fingerprint pattern in future work. All specimens were sanded with #1000 grit sandpaper to remove unintended microscopic asperities.

Throughout this paper, specimens are labeled using a two-letter code. The first letter indicates the type of specimen, where “C” represents circular specimens, and “R” represents rectangular specimens. The second letter corresponds to the size of the surface features. For instance, “C3” denotes a circular specimen with a 3.0 mm diameter, and “R4” refers to a rectangular specimen with a 4.0 mm wavelength.

## 3. Methods

### 3.1. Magnitude Estimation Method to Collect Roughness Perceived from Specimens

To collect subjective roughness values, the psychophysical method of magnitude estimation was employed. This method is designed to quantify the relationship between physical stimuli and the perceptions they elicit [63,64] and has been adopted by many researchers for investigating humans’ roughness perception (e.g., [65]).

Magnitude estimation allows participants to assign numerical values to the perceived intensity of a stimulus, specifically surface roughness in this study. A reference specimen is designated as a baseline value, and participants then rate the perceived magnitude of other stimuli relative to this reference. Participants indicate how many times stronger the stimulus intensity feels compared to the reference.

In this study, subjective roughness data were acquired following the procedure outlined below:

1. A single specimen, labeled R3, was designated as the reference stimulus and assigned a roughness value of 1.0. Participants could freely touch this reference specimen while evaluating other specimens.
2. Only pressing motions were permitted; sliding motions were strictly prohibited. This was enforced through clear instructions and continuous monitoring by the experimenters. Any deviation from the instructed motion was promptly addressed with a verbal reminder or, if necessary, by repeating the trial. The level of pressing force was not instructed in order to encourage natural interaction.
3. To ensure that roughness estimations were based solely on tactile perception, participants wore glasses with textured stickers to block their vision.
4. After touching each test specimen with their index finger, participants rated its subjective roughness relative to the reference specimen.
5. This procedure was repeated until all randomly presented test specimens had been evaluated within a single session.
6. Each participant completed three separate sessions to ensure data reliability.

Data were collected from ten participants (mean age: 26.0 years), all of whom provided written informed consent prior to the study. The procedure was approved by the institutional review board of Tokyo Metropolitan University, Hino Campus (approval number: R6-009, approved on 19 April 2024).

The final subjective roughness values were determined through a two-step process. First, the median value of the three repetitions was calculated for each specimen and participant. Then, the geometric mean of these median values was computed across all participants for each specimen.

### 3.2. Acquisition of Contact Images

For the image acquisition procedure, the workbench in Section 2.1 was used. The steps for image acquisition were as follows:

1. Each specimen was mounted on the workbench, and a pressing force of 1 N was applied. An image of the contact area was then captured.
2. The pressing force was increased to 2 N, and a second image of the contact area was taken.
3. The pressing force was further increased to 3 N, and a third image of the contact area was taken. This force is substantially greater than the pressing force typically exerted by humans during surface roughness exploration [66,67].
4. The specimen was replaced, and the imaging process was repeated for the next specimen.
5. This procedure was performed 10 times for each specimen to capture multiple images, accounting for slight variations across trials.

**Figure 5:**
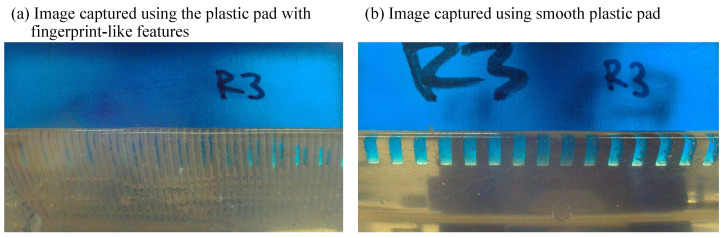
Pictures taken from the workbench. Specimen R3 was used as the sample.

**Figure 6:**
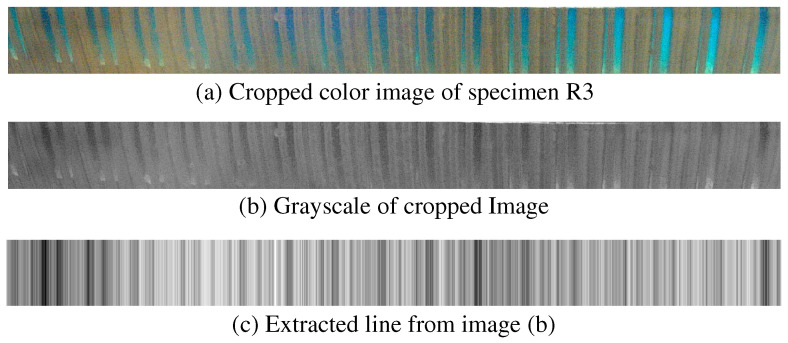
Image processing process. (**a**) Cropped version of original picture. (**b**) Grayscale version of image (**a**). (**c**) A horizontal line extracted from the middle of image (**b**).

Figure 5 presents a sample image of the contact area. To isolate the contact area, each original image was cropped to a width of 1900 pixels and a height of 150 pixels, as shown in Figure 6a. Following cropping, the images were converted to grayscale, as illustrated in Figure 6b. Next, the middle portion of the image was extracted horizontally, as depicted in Figure 6c. This process was applied to all images obtained from the specimens. Finally, the extracted gray-level intensity was transformed into an amplitude spectrum using the Fourier transform. In total, 420 images were processed (14 specimens × 3 force levels × 10 repetitions) for each of the two contact pads. All image processing was performed using MATLAB (2024a, MathWorks, Inc., Natick, MA, USA).

We used spatial frequencies ranging from 0.1 mm^−1^ (corresponding to 10 mm) to 1.0 mm^−1^ (corresponding to 1 mm), comprising 45 discrete frequency components. These data points served as predictors in the analysis. To mitigate the effects of environmental brightness on the captured images, the spectral amplitudes were normalized to ensure that the area under the spectral curve remained consistent across different images. Notably, this process does not alter the spectral profile or peak positions.

### 3.3. Prediction of Roughness Perception from Weighted Spatial Spectra of Contact Area

In this study, Partial Least Squares (PLS) regression was employed as the primary analysis tool. PLS regression is a statistical learning method that relates a set of predictors to one or more response variables by identifying latent variables or components that predominantly determine the relationships between the predictors and response variables [68,69,70,71,72]. When there is only one response variable, the method is referred to as PLS1. For the computation of PLS1, we used the plsregress function of MATLAB (2024a, MathWorks Inc., Natick, MA, USA) that uses the SIMPLS algorithm [73,74].

The analysis aims to establish a relationship between the predictor matrix $X\in R^{n\times p}$ and the response vector $y\in R^{n\times 1}$. Here, X represents the matrix of *p* independent variables (predictors), and *n* denotes the number of samples, given by n=140 (14 specimens × 10 repetitive measurements). In this study, the predictors correspond to the spatial spectral components computed from the contact area images, with *p* denoting the number of components used for prediction. The response vector y consists of the subjective roughness values of the specimens, obtained through the magnitude estimation method described in Section 3.1.

In this study, the response variable is univariate ($y\in R^{n\times 1}$); therefore, the vector of covariances between X and y is computed as follows:

(1) $$s_{a}=X_{a}^{T}y_{a}.$$

Here, $s_{a}\in R^{p\times 1}$ represents the covariance vector. We use $X_{a}$ and $y_{a}$ (a=1,…,A), instead of X and y, respectively. *A* denotes the total number of extracted components. In the algorithm of PLS, we start from a=1, and $X_{1}=X$ and $y_{1}=y$.

Next, the weight vector is extracted:

(2) $$r_{a}=\frac{s_{a}}{∥s_{a}∥}.$$

where $r_{a}\in R^{p\times 1}$ is the weight vector for the *a*th PLS component. The notation ∥•∥ represents the $L_{2}$-norm.

The score vector is then computed as follows:

(3) $$t_{a}=X_{a}r_{a}.$$

Here, $t_{a}\in R^{n\times 1}$ represents the score vector of $X_{a}$, which corresponds to the projection of the predictor matrix $X_{a}$ onto the weight vector $r_{a}$.

Next, the loading vector $p_{a}\in R^{p\times 1}$ is computed as follows:

(4) $$p_{a}=\frac{X_{a}^{T}t_{a}}{t_{a}^{T}t_{a}},$$

which quantifies the contribution of each predictor to the latent factor $t_{a}$.

Next, the regression coefficient for *a*-th component $b_{a}$ is computed from the following:

(5) $$b_{a}=\frac{t_{a}^{T}y_{a}}{t_{a}^{T}t_{a}}.$$

The above computation is performed for the *a*th component. To compute the next component, $X_{a}$ and $y_{a}$ are deflated to ensure that it remains uncorrelated with the previously extracted components:

(6) $$\begin{array}{c} X_{a+1}=X_{a}-t_{a}p_{a}^{T} \end{array}$$

(7) $$\begin{array}{c} y_{a+1}=y_{a}-b_{a}t_{a}. \end{array}$$

The final regression model is expressed as follows:

(8) $$\hat{y}=X\sum_{a=1}^{A}b_{a}r_{a}+\bar{y}$$

where $\hat{y}$ and $\bar{y}$ represent the predicted values and the mean of the response variable *y*, respectively. Note that $b_{a}$ and $r_{a}$ are determined using the training dataset. This formula can then be applied to predict y values for the test dataset X.

The predictors in this study were the amplitudes of spatial frequencies ranging from 0.1 to 1.0 mm^−1^, comprising 45 discrete components. When roughness prediction was based on images obtained under a pressing force of either 1 N, 2 N, or 3 N, the number of predictors was p=45. When combining datasets from all three pressing forces (1 N, 2 N, and 3 N), the number of predictors tripled to 135 (p=135).

To determine the optimal number of principal components (*A*) for the PLS model, leave-one-out cross-validation was employed. Leave-one-out cross-validation is a special case of cross-validation where the number of folds equals the number of instances in the dataset. Thus, only a single sample is held aside for validation in each iteration.

These processes—namely, the establishment and cross-validation of the PLS models—were conducted separately for images captured using pads with and without fingerprint-like ridges. This allowed for a direct comparison of performance between the two types of sensor pads.

### 3.4. Performance Indices

We employ two performance indices: root mean squared error (RMSE) and overlap coefficient (OVL).

For the RMSE calculation, we refer to the geometric mean of participants’ estimates. For each sample, the error between the predicted value and the participants’ mean estimate was computed, and the RMSE was then derived from these errors.

However, participants’ estimates naturally vary across individuals. Thus, evaluating only the RMSE may not fully capture the alignment between predicted and actual distributions. To address this, we also employ the overlap coefficient (OVL), a metric that quantifies the similarity between two probability distributions by measuring the shared area under their curves [75,76]. This measure is particularly useful for comparing a model’s predicted probability distribution with the actual data distribution.

The OVL is defined as follows:

(9) $$OVL=\int_{-\infty }^{\infty }\min\left(f_{1}\left(x\right),f_{2}\left(x\right)\right)\;dx,$$

where $f_{1}\left(x\right)$ and $f_{2}\left(x\right)$ are the probability density functions of the two distributions. The OVL value ranges from 0 to 1, where 1 indicates perfect overlap (identical distributions), and 0 indicates no overlap (completely distinct distributions). For the calculation of OVL values, the distributions of perceived and estimated roughness were approximated as normal distributions. This assumption was made due to the limited sample size (10 samples per distribution), which made it challenging to reliably determine the actual distribution type.

## 4. Results

**Figure 7:**
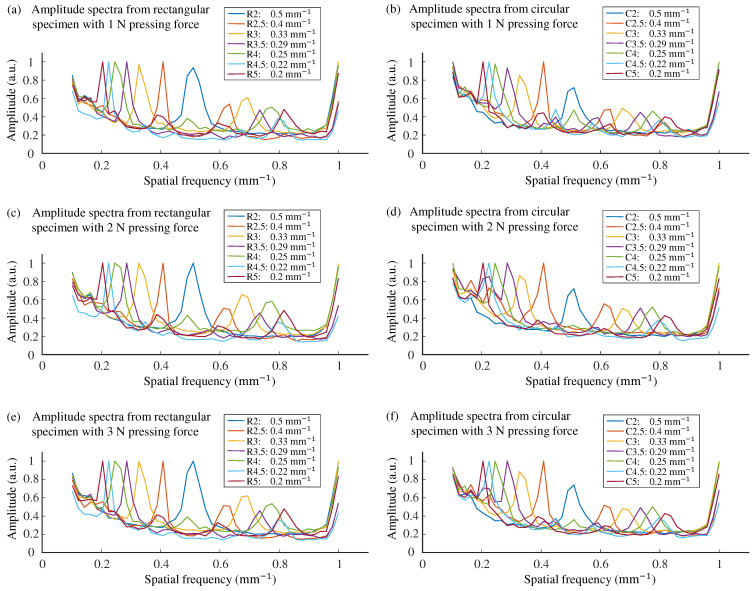
Amplitude spectra from all specimens and all pressing forces. Each curve represents the average across 10 repetition for one specimen.

Figure 7 shows the normalized amplitude spectra of the contact area by different roughness specimens and contact force levels. The amplitude spectrum contains two distinct peaks: one at 1 mm^−1^, representing the frequency of the fingerprint-like ridges on the sensor pad, and another corresponding to the wavelength of the specimen.

**Figure 8:**
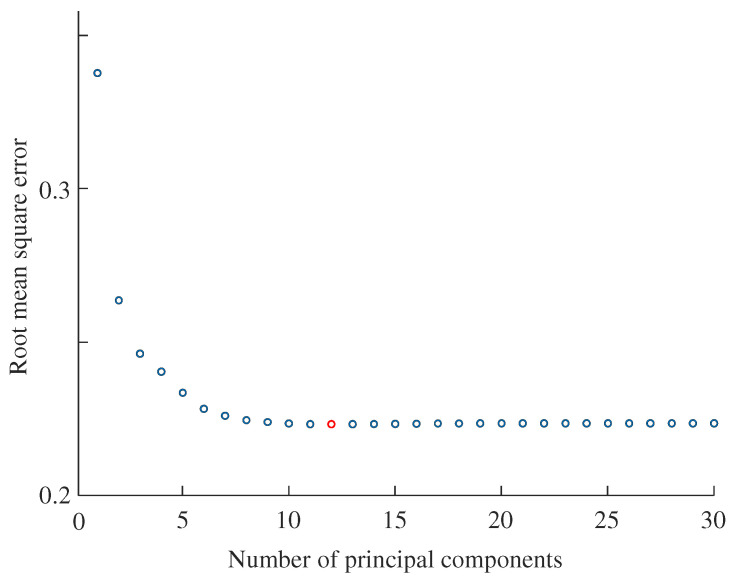
Root mean square errors from leave-one-out cross-validation results, showing the variation with the number of principal components. One hundred and thirty-five predictors (p=135) were used by combining datasets from 1 N, 2 N, and 3 N pressing forces. The error reached its minimum value (red circle) with twelve principal components (A=12). The RMSE values for 11, 12, and 13 components were 0.22330, 0.22326, and 0.22328, respectively.

Based on cross-validation, the number of principal components was determined for each pressing force condition. For the contact pad with fingerprint-like ridges, the lowest RMSE values were obtained when the number of principal components (*A*) was eight, eight, ten, and twelve for the 1 N, 2 N, 3 N, and combined conditions, respectively. Figure 8 shows the case for the combined condition, where the RMSE reached its minimum value with twelve components. Beyond this point, RMSE values slightly increased. For the smooth contact pad, the optimal number of principal components was 11, 10, 12, and 15 for the 1 N, 2 N, 3 N, and combined conditions, respectively.

Table 1, Table 2 and Table 3 show the results for the sensor pad with fingerprint-like ridges.

Table 1 presents the results of the magnitude estimation method and predictions using PLS regression. The first column lists the labels of the specimens. The second column shows the geometric mean and standard deviation of the magnitude estimates across the participants for each specimen. Notably, larger ridge and groove sizes resulted in higher roughness ratings, with C5 and R5 exhibiting the highest perceived roughness in their respective types. The third column displays the mean roughness scores predicted by the PLS model using datasets obtained under the pressing force of 1 N and standard deviation for each specimen. Similarly, the fourth, fifth, and sixth columns display the predicted values using datasets obtained under pressing forces of 2 N, 3 N, and a combination of all pressing forces.

Table 2 presents the OVL coefficients quantifying the overlap between the distributions of predicted roughness scores and participants’ estimates. The first column lists the specimen labels. The second column shows the OVL values for predictions made under a 1 N pressing force. Similarly, the third, fourth, and fifth columns present the OVL values for datasets obtained under 2 N, 3 N, and a combination of all pressing forces. It is noted that as we did not collect the participants’ responses for R3, the OVL values for R3 were not calculated.

**Figure 9:**
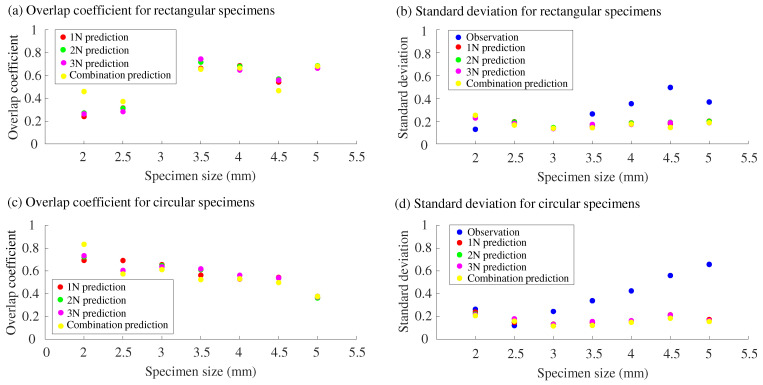
Results using the pad with fingerprint-like features. Overlap (OVL) coefficients ((**a**) rectangular, (**c**) circular) and standard deviations of human estimates and PLS predictions ((**b**) rectangular, (**d**) circular) for each specimen.

Figure 9 presents a visualization of the data in Table 2, alongside the standard deviations of human estimates and PLS predictions listed in Table 1. The trends in OVL values differ noticeably between the two types of roughness scales. For the rectangular scale, as shown in Figure 9a, OVL values largely remained around 0.6 for specimen sizes of 3.5 mm or greater. In contrast, for the circular scale (Figure 9c), OVL values consistently decreased as specimen size increased.

This difference can be attributed to two main factors. First, for the circular specimens (Figure 9d), the standard deviations of human roughness estimates increased monotonically with specimen size. Second, as shown in Table 1, the roughness scores for rectangular specimens were estimated with relatively high accuracy for larger specimen sizes, such as R4, R4.5, and R5. In contrast, the roughness scores for circular specimens were predicted with relatively low accuracy, particularly for C4.5 and C5. As a result, for the circular specimens, both the central value and spread of the distributions diverged between the human estimates and the PLS predictions at larger specimen sizes.

Similarly, Table 3 presents the RMSE for each specimen under different pressing forces. We compared the combined condition, which exhibited the lowest mean RMSE value of 0.21, with the other pressing force conditions using a signed-rank test (signrank function, MATLAB 2024a) with a Bonferroni adjustment factor of 3. The RMSE values for the combined condition were significantly lower than those for the individual conditions: combined vs. 1 N: W=6, $p=4.8\times 10^{-3}$; combined vs. 2 N: W=14, $p=7.2\times 10^{-4}$; combined vs. 3 N: W=0, $p=1.2\times 10^{-4}$.

Table 4, Table 5 and Table 6 show the results for the smooth pad without fingerprint-like features. Table 4 presents the roughness values estimated from images. As listed in Table 5, the mean and standard deviation of the OVL coefficients were 0.59±0.17, 0.59±0.16, 0.55±0.16, and 0.57±0.19 for the 1 N, 2 N, 3 N, and combined conditions, respectively. These values are comparable to those obtained using the fingerprinted pad, as listed in Table 2. Table 6 shows the RMSEs between the perceived roughness values and those predicted using the smooth pad. Even with the smooth pad, combining images captured under three different levels of pressing force led to improved prediction accuracy.

As discussed in Section 2.2, we aimed to compare the performance of the two types of sensor pads. Under the combined-force condition, the RMSE values obtained using the smooth pad (average: 0.13) were significantly lower than those obtained with the fingerprinted pad (average: 0.21) (signed rank test, W=6, p<0.0017). This result indicates that the presence of fingerprint patterns slightly degrades roughness estimation performance, with an RMSE difference of approximately 0.08.

## 5. Discussion

Here, we evaluate the prediction accuracy of the pad with fingerprint-like structures, using RMSE and OVL values as performance metrics.

We first compare the four pressing force conditions (1 N, 2 N, 3 N, and the combined condition). The mean RMSE values across all specimens were 0.26, 0.25, 0.25, and 0.21 for 1 N, 2 N, 3 N, and the combined condition, respectively. Statistically, the combined condition exhibited lower RMSE values than the individual force conditions; however, the differences in mean RMSE values were small, ranging from 0.04 to 0.05, which is not practically significant. The mean OVL factors across all specimens were 0.56 for all conditions. Since these indices did not show distinct differences among the four pressing force conditions, we do not currently have strong evidence to recommend a specific pressing force.

The predictive accuracy of the model was evaluated based on RMSE values for 14 distinct specimens. The RMSE values for the combined-force condition ranged from 0.15 to 0.35, which were smaller than or comparable to the standard deviations of perceived roughness (0.12–0.64) for most specimens, as listed in Table 1. Under the combined force condition, the largest RMSE values were recorded for R2, R2.5, and C5, with values of 0.35, 0.34, and 0.31, respectively. These results suggest that the estimation errors were generally small compared to the variability in human roughness perception; however, for certain roughness specimens, the estimation errors remained relatively large.

Although a specific criterion for classifying the strength of overlap—such as strong, moderate, or weak—using the overlap coefficient is not well established in the literature, the OVL factors of 0.56 fairly suggest moderate overlap between two probabilistic density functions.

The analysis identified three specimens—R2, R2.5, and C5—that had OVL values below 0.5 across all pressing force conditions. This is primarily due to the extremely small or large standard deviations of perceived roughness for R2, R2.5, and C5, which were 0.13, 0.18, and 0.64, respectively. As a result, the distributions of perception and prediction exhibited minimal overlap. These findings suggest that our roughness prediction method does not fully capture the individual fluctuations in perception that are inherent to human tactile sensation.

The prediction accuracy was higher when using the pad without fingerprint-like structures. This suggests that the fingerprints may act as visual noise during the imaging of the contact area. Nonetheless, the average difference in RMSE between the two contact pads was 0.08, which is smaller than the standard deviation of human roughness estimates listed in Table 1 (ranging from 0.13 to 0.64). One possible reason is that the fingerprint pattern on the pad used in this study is approximately twice the size of actual human fingerprints, and thus, downsizing the pattern could potentially lead to improved accuracy. If such an attempt proves unsuccessful, another approach could be to use separate contact pads for sensing macroscopic and microscopic roughness. Although our goal is to integrate both functions into a single tactile sensor, it may be more effective to optimize each pad for a specific roughness scale. In this case, a pad with fingerprint patterns could be used for microscopic roughness sensing (to enhance vibration transmission during sliding), while a pad without fingerprint patterns could be used for macroscopic roughness estimation based on contact imaging.

During the magnitude estimation experiments, we observed that the results for circular specimens differed from our expectations. We had anticipated that circular specimens would feel smoother (i.e., less rough) than rectangular specimens as the latter have sharp edges at the ends of ridges. However, the pressing experiment revealed that participants perceived rectangular specimens as less rough than circular specimens of the same spatial period (e.g., R2 compared to C2).

This discrepancy may be explained from the viewpoint of groove widths. Many studies have shown that the roughness perception of grating scales with macroscopic features is primarily determined by their groove widths [77,78,79,80,81]. Larger groove widths lead to greater perceived roughness, provided that the ridge height is sufficient to prevent the penetrated skin from reaching the bottom of the groove. The rectangular gratings used in this study had a ridge-to-groove width ratio of 1:1; thus, for example, the groove width of R3 was 1.5 mm. In contrast, for C3 (circular gratings with a diameter of 3 mm), the net groove width—defined as the width where the finger pad does not contact the specimen’s surface—was expected to be greater than 1.5 mm under natural pressing forces. Hence, in our study, circular gratings were perceived as rougher than rectangular ones.

As shown in Figure 9b,d, human roughness perception becomes more variable as the specimen size increases. This is likely a natural outcome consistent with Weber’s law: when the stimulus magnitude is larger, the discrimination threshold in perception also tends to increase. In other words, for larger specimens associated with higher perceived roughness, the standard deviation of human responses also increases. In contrast, the output variability of our sensor system remained nearly constant across specimen sizes, with a standard deviation of approximately 0.2. This suggests that mimicking human spatial coding alone is not sufficient to fully replicate the variability observed in human perceptual processing.

Here, we describe the limitations of this study.

Although we tested two different types of roughness scales—rectangular and circular—to investigate the generalizability of the method, additional types of scales should be explored. For instance, random grating scales [57,82] are used to study human roughness perception in scenarios more representative of daily life.

Furthermore, the fingerprint-like ridges of the compliant pad need to be downsized to match the size of human fingerprints, and roughness specimens with smaller spatial periods than those tested in this study should be examined in future research.

Our method is based on the contact area of the surface; however, SAI units are known to respond to strain in the skin tissue [27,28,29]. Thus, our approach should incorporate mechanical information, such as surface deformation, rather than relying solely on contact area. We initially expected that combining images captured at different pressing forces would provide insights into the penetration depth of the surface material into the specimens’ grooves, thereby improving prediction accuracy. However, the results indicated only a slight advantage in utilizing images from multiple pressing forces. Future research should explore more effective methods to leverage these images.

Another potential area for future research is minimizing the contact pad as its current size is substantially larger than that of a human finger pad. It remains unclear whether the pad needs to match the size of a human finger pad. Nonetheless, downsizing is generally advantageous for industrial applications, provided that performance is maintained.

## 6. Conclusions

This study explores the potential of predicting macroscopic roughness using the contact area of the sensor body as a predictor. Previous approaches primarily focused on accurately representing topology of roughened surfaces. In contrast, we aimed to predict roughness as perceived by humans, mimicking the principle of spatial coding in human roughness perception. To achieve this, we analyzed the spatial spectra of contact images captured between a transparent compliant probe and roughness specimens.

The findings revealed that estimated macroscopic roughness largely aligns with human subjective roughness magnitudes, suggesting that roughness estimation based on spatial frequencies in contact images correlates well with human perception of surface texture. For future research, expanding the range of textural shapes and refining prediction models would be valuable to enhance accuracy and applicability.

## Figures and Tables

**Table 1:** Results using the pad with fingerprint-like features. Perceived and estimated roughness values under four different predictor datasets. Values are presented as mean ± standard deviation.

Specimen	Perceived	Prediction			
	Roughness	Dataset 1 N	Dataset 2 N	Dataset 3 N	Dataset Combination
R2	0.37±0.13	0.80±0.25	0.75±0.23	0.76±0.23	0.62±0.25
R2.5	0.66±0.18	1.03±0.19	1.03±0.19	1.04±0.18	0.96±0.16
R3	1.00	1.13±0.14	1.12±0.14	1.12±0.13	1.10±0.13
R3.5	1.14±0.26	1.27±0.15	1.25±0.17	1.24±0.17	1.25±0.14
R4	1.39±0.35	1.33±0.18	1.33±0.18	1.31±0.17	1.35±0.17
R4.5	1.48±0.49	1.46±0.18	1.47±0.19	1.49±0.19	1.50±0.14
R5	1.53±0.37	1.41±0.20	1.43±0.20	1.44±0.18	1.53±0.18
C2	0.70±0.26	0.90±0.24	0.86±0.22	0.86±0.21	0.78±0.21
C2.5	1.23±0.12	1.13±0.15	1.10±0.16	1.09±0.18	1.08±0.16
C3	1.29±0.24	1.18±0.13	1.18±0.13	1.17±0.13	1.19±0.11
C3.5	1.39±0.33	1.23±0.14	1.27±0.15	1.29±0.15	1.29±0.12
C4	1.41±0.43	1.29±0.15	1.32±0.15	1.32±0.16	1.35±0.15
C4.5	1.65±0.55	1.46±0.21	1.48±0.21	1.46±0.21	1.50±0.18
C5	1.72±0.64	1.35±0.17	1.38±0.16	1.38±0.16	1.46±0.15

**Table 2:** Results using the pad with fingerprint-like features. Overlap coefficient (OVL) calculated between the distributions of magnitude estimates and predicted values under different pressing forces.

Specimen	Overlap Coefficient (OVL)			
	Dataset 1 N	Dataset 2 N	Dataset 3 N	Dataset Combination
R2	0.24	0.27	0.27	0.46
R2.5	0.32	0.32	0.29	0.38
R3	−	−	−	−
R3.5	0.67	0.72	0.75	0.66
R4	0.69	0.69	0.65	0.67
R4.5	0.55	0.57	0.56	0.47
R5	0.68	0.68	0.66	0.67
C2	0.69	0.72	0.73	0.84
C2.5	0.71	0.62	0.62	0.59
C3	0.66	0.65	0.64	0.61
C3.5	0.57	0.62	0.62	0.53
C4	0.52	0.52	0.55	0.52
C4.5	0.55	0.54	0.54	0.50
C5	0.38	0.37	0.38	0.38
Rectangular:				
Mean ± S.D.	0.52±0.20	0.54±0.20	0.53±0.20	0.55±0.13
Circular:				
Mean ± S.D.	0.58±0.12	0.58±0.11	0.58±0.11	0.57±0.14
Overall:				
Mean ± S.D.	0.56±0.15	0.56±0.15	0.56±0.16	0.56±0.13

**Table 3:** Results using the pad with fingerprint-like features. Root mean squared error (RMSE) calculated based on predicted values and magnitude estimates under different pressing forces.

Specimen	Root Mean Squared Error (RMSE)			
	Dataset 1 N	Dataset 2 N	Dataset 3 N	Dataset Combination
R2	0.49	0.45	0.45	0.35
R2.5	0.42	0.42	0.42	0.34
R3	0.19	0.18	0.18	0.17
R3.5	0.19	0.20	0.20	0.18
R4	0.19	0.19	0.19	0.18
R4.5	0.18	0.19	0.19	0.15
R5	0.23	0.22	0.21	0.18
C2	0.31	0.27	0.26	0.22
C2.5	0.18	0.21	0.22	0.21
C3	0.17	0.17	0.17	0.15
C3.5	0.21	0.20	0.19	0.16
C4	0.19	0.17	0.18	0.16
C4.5	0.28	0.27	0.28	0.23
C5	0.42	0.38	0.38	0.31
Mean ± S.D.	0.26±0.11	0.25±0.09	0.25±0.10	0.21±0.07

**Table 4:** Results using the smooth pad. Perceived and estimated roughness values under four different predictor datasets. Values are presented as mean ± standard deviation. The column for perceived roughness is identical to that in Table 1.

Specimen	Perceived	Prediction			
	Roughness	Dataset 1 N	Dataset 2 N	Dataset 3 N	Dataset Combination
R2	0.37±0.13	0.52±0.22	0.58±0.22	0.51±0.15	0.45±0.14
R2.5	0.66±0.18	0.85±0.20	0.88±0.19	0.91±0.16	0.78±0.16
R3	1.00	1.08±0.13	1.12±0.12	1.09±0.12	1.04±0.11
R3.5	1.14±0.26	1.21±0.13	1.27±0.13	1.26±0.12	1.21±0.11
R4	1.39±0.35	1.30±0.15	1.30±0.14	1.31±0.13	1.34±0.12
R4.5	1.48±0.49	1.45±0.15	1.46±0.14	1.51±0.12	1.47±0.11
R5	1.53±0.37	1.45±0.16	1.45±0.20	1.49±0.15	1.49±0.12
C2	0.70±0.26	0.73±0.13	0.71±0.17	0.70±0.11	0.71±0.10
C2.5	1.23±0.12	1.16±0.13	1.13±0.12	1.07±0.13	1.18±0.10
C3	1.29±0.24	1.25±0.09	1.19±0.11	1.18±0.13	1.25±0.09
C3.5	1.39±0.33	1.32±0.10	1.26±0.10	1.29±0.11	1.33±0.09
C4	1.41±0.43	1.42±0.10	1.43±0.13	1.41±0.12	1.43±0.09
C4.5	1.65±0.55	1.61±0.15	1.59±0.14	1.58±0.13	1.63±0.13
C5	1.72±0.64	1.60±0.13	1.59±0.15	1.64±0.13	1.66±0.12

**Table 5:** Results using the smooth pad. Overlap coefficient (OVL) calculated between the distributions of magnitude estimates and predicted values under different pressing forces.

Specimen	Overlap Coefficient (OVL)			
	Dataset 1 N	Dataset 2 N	Dataset 3 N	Dataset Combination
R2	0.62	0.51	0.63	0.77
R2.5	0.61	0.54	0.44	0.71
R3	−	−	−	−
R3.5	0.66	0.62	0.59	0.58
R4	0.60	0.58	0.54	0.53
R4.5	0.48	0.46	0.41	0.39
R5	0.60	0.69	0.58	0.50
C2	0.66	0.78	0.60	0.58
C2.5	0.79	0.68	0.52	0.81
C3	0.57	0.61	0.65	0.56
C3.5	0.48	0.46	0.50	0.45
C4	0.38	0.47	0.45	0.36
C4.5	0.43	0.42	0.39	0.41
C5	0.36	0.39	0.34	0.33
Rectangular:				
Mean ± S.D.	0.65±0.16	0.63±0.18	0.60±0.19	0.64±0.20
Circular:				
Mean ± S.D.	0.53±0.16	0.55±0.15	0.49±0.11	0.50±0.17
Overall:				
Mean ± S.D.	0.59±0.17	0.59±0.16	0.55±0.16	0.57±0.19

**Table 6:** Results using the smooth pad. Root mean squared error (RMSE) calculated based on predicted values and magnitude estimates under different pressing forces.

Specimen	Root Mean Squared Error (RMSE)			
	Dataset 1 N	Dataset 2 N	Dataset 3 N	Dataset Combination
R2	0.26	0.30	0.20	0.16
R2.5	0.28	0.29	0.30	0.20
R3	0.15	0.17	0.15	0.12
R3.5	0.15	0.18	0.17	0.13
R4	0.18	0.17	0.15	0.13
R4.5	0.15	0.14	0.12	0.11
R5	0.17	0.21	0.15	0.12
C2	0.13	0.17	0.11	0.10
C2.5	0.15	0.15	0.20	0.12
C3	0.10	0.15	0.17	0.10
C3.5	0.12	0.16	0.15	0.11
C4	0.10	0.13	0.12	0.09
C4.5	0.15	0.15	0.14	0.13
C5	0.18	0.20	0.15	0.14
Mean ± S.D.	0.16±0.05	0.18±0.05	0.16±0.05	0.13±0.03

## Data Availability

Dataset available on request from the authors.

## References

[1] Kawabata S., Niwa M., Raheel M. (1996). Objective measurement of fabric hand. Modern Textile Characterization Methods.

[2] Peck J., Childers T.L. (2003). Individual differences in haptic information processing: The “need for touch” scale. J. Consum. Res..

[3] Chen X., Barnes C.J., Childs T.H.C., Henson B., Shao F. (2009). Materials’ tactile testing and characterization for consumer products’ affective packaging design. Mater. Des..

[4] Klöcker A., Arnould C., Penta M., Thonnard J.L. (2012). Rasch-built measure of pleasant touch through active fingertip explorations. Front. Neurorobot..

[5] Kadoya Y., Khan M.S.R., Watanapongvanich S., Fukada M., Kurita Y., Takahashi M., Machida H., Yarimizu K., Kimura N., Sakurai H. (2022). Consumers’ willingness to pay for tactile impressions: A study using smartphone covers. IEEE Access.

[6] Richardson B.A., Kuchenbecker K.J. (2020). Learning to predict perceptual distributions of haptic adjectives. Front. Neurorobot..

[7] Bicchi A., Schilingo E.P., De Rossi D. (2000). Haptic discrimination of softness in teleoperation: The role of the contact area spread rate. IEEE Trans. Robot. Autom..

[8] Scilingo E.P., Bianchi M., Grioli G., Bicchi A. (2010). Rendering softness: Integration of kinesthetic and cutaneous information in a haptic device. IEEE Trans. Haptics.

[9] Asaga E., Takemura K., Maeno T., Ban A., Toriumi M. (2013). Tactile evaluation based on human tactile perception mechanism. Sens. Actuators A Phys..

[10] Hashim I.H.M., Kumamoto S., Takemura K., Maeno T., Okuda S., Mori Y. (2017). Tactile evaluation feedback system for multi-layered structure inspired by human tactile perception mechanism. Sensors.

[11] Saito N., Matsumori K., Kazama T., Sakaguchi S., Okazaki R., Arakawa N., Okamoto S. (2023). Skin quality sensor to evaluate vibration and friction generated when sliding over skins. Int. J. Cosmet. Sci..

[12] Ahirwar M., Behera B.K. (2021). Fabric hand research translates senses into numbers—A review. J. Text. Inst..

[13] Bensmaïa S.J. (2009). Texture from touch. Scholarpedia.

[14] Okamoto S., Nagano H., Yamada Y. (2013). Psychophysical dimensions of tactile perception of textures. IEEE Trans. Haptics.

[15] Wettels N., Loeb G.E. Haptic feature extraction from a biomimetic tactile sensor: Force, contact location and curvature. Proceedings of the IEEE International Conference on Robotics and Biomimetics.

[16] Culbertson H., Kuchenbecker K.J. (2017). Importance of matching physical friction, hardness, and texture in creating realistic haptic virtual surfaces. IEEE Trans. Haptics.

[17] Ding S., Pan Y., Tong M., Zhao X. (2017). Tactile perception of roughness and hardness to discriminate materials by friction-induced vibration. Sensors.

[18] Mukaibo Y., Shirado H., Konyo M., Maeno T. Development of a texture sensor emulating the tissue structure and perceptual mechanism of human fingers. Proceedings of the IEEE International Conference on Robotics and Automation.

[19] Kim S.J., Choi J.Y., Moon H., Choi H.R., Koo J.C. (2020). Biomimetic hybrid tactile sensor with ridged structure that mimics human fingerprints to acquire surface texture information. Sens. Mater..

[20] Kim K., Sim M., Lim S.H., Kim D., Lee D., Shin K., Moon C., Choi J.W., Jang J.E. (2021). Tactile avatar: Tactile sensing system mimicking human tactile cognition. Adv. Sci..

[21] Shirakawa K., Tanaka Y., Hashimoto M., Watarai E., Igarashi T. (2021). Wearable Artificial Fingers with Skin Vibration and Multi-Axis Force Sensors. IEEE Trans. Haptics.

[22] Ke A., Huang J., Chen L., Gao Z., Han J., Wang C., Zhou J., He J. (2019). Fingertip Tactile Sensor with Single Sensing Element Based on FSR and PVDF. IEEE Sens. J..

[23] Liu W., Yu P., Gu C., Cheng X., Fu X. (2017). Fingertip Piezoelectric Tactile Sensor Array for Roughness Encoding Under Varying Scanning Velocity. IEEE Sens. J..

[24] Yanwari M.I., Okamoto S. (2024). Healing function for abraded fingerprint ridges in tactile texture sensors. Sensors.

[25] Hollins M., Bensmaïa S.J. (2007). The coding of roughness. Can. J. Exp. Psychol..

[26] Hollins M., Bensmaïa S., Roy E. (2002). Vibrotaction and texture perception. Behav. Brain Res..

[27] Johansson R.S., Vallbo B.Å. (1983). Tactile sensory coding in the glabrous skin of the human hand. Trends Neurosci..

[28] Phillips J.R., Johnson K.O. (1981). Tactile spatial resolution. III. A continuum mechanics model of skin predicting mechanoreceptor responses to bars, edges, and gratings. J. Neurophysiol..

[29] Lesniak D.R., Gerling G.J. (2009). Predicting SA-I mechanoreceptor spike times with a skin-neuron model. Math. Biosci..

[30] Corniani G., Saal H.P. (2020). Tactile innervation densities across the whole body. J. Neurophysiol..

[31] Johansson R.S. (1978). Tactile sensibility in the human hand: Receptive field characteristics of mechanoreceptive units in the glabrous skin area. J. Physiol..

[32] Blake D.T., Hsiao S.S., Johnson K.O. (1997). Neural Coding Mechanisms in Tactile Pattern Recognition: The Relative Contributions of Slowly and Rapidly Adapting Mechanoreceptors to Perceived Roughness. J. Neurosci..

[33] Connor C.E., Johnson K.O. (1992). Neural Coding of Tactile Texture: Comparison of Spatial and Temporal Mechanisms for Roughness Perception. J. Neurosci..

[34] Weber A.I., Saal H.P., Lieber J.D., Cheng J.W., Manfredi L.R., Dammann J.F.I., Bensmaïa S.J. (2013). Spatial and temporal codes mediate the tactile perception of natural textures. Proc. Natl. Acad. Sci. USA.

[35] Sun Q., Okamoto S., Akiyama Y., Yamada Y. (2022). Multiple Spatial Spectral Components of Static Skin Deformation for Predicting Macroscopic Roughness Perception. IEEE Trans. Haptics.

[36] Okamoto S., Oishi A. (2020). Relationship between spatial variations in static skin deformation and perceived roughness of macroscopic surfaces. IEEE Trans. Haptics.

[37] Lamb G.D. (1983). Tactile discrimination of textured surfaces: Psychophysical performance measurements in humans. J. Physiol..

[38] Meftah E.M., Belingard L., Chapman C.E. (2000). Relative effects of the spatial and temporal characteristics of scanned surfaces on human perception of tactile roughness using passive touch. Exp. Brain Res..

[39] Hollins M., Rinser S.R. (2000). Evidence for the Duplex Theory of Tactile Texture Perception. Atten. Percept. Psychophys..

[40] Tymms C., Zorin D., Gardne E.P. (2018). Tactile perception of the roughness of 3D-printed textures. J. Neurophysiol..

[41] Arvidsson M., Ringstad L., Skedung L., Duvefelt K., Rutland M.W. (2017). Feeling fine—The effect of topography and friction on perceived roughness and slipperiness. Biotribology.

[42] Johnson M.K., Adelson E.H. Retrographic sensing for the measurement of surface texture and shape. Proceedings of the IEEE Conference on Computer Vision and Pattern Recognition.

[43] Maheshwari V., Saraf R.F. (2006). High-Resolution Thin-Film Device to Sense Texture by Touch. Science.

[44] Li R., Adelson E.H. Sensing and Recognizing Surface Textures Using a GelSight Sensor. Proceedings of the IEEE Conference on Computer Vision and Pattern Recognition.

[45] Ito Y., Kim Y., Nagai C., Obinata G. (2012). Vision-Based Tactile Sensing and Shape Estimation Using a Fluid-Type Touchpad. IEEE Trans. Autom. Sci. Eng..

[46] Saga S., Kajimoto H., Tachi S. (2007). High-resolution tactile sensor using the deformation of a reflection image. Sens. Rev..

[47] Cummins H., Waits W.J., McQuitty J.T. (1941). The breadths of epidermal ridges on the finger tips and palms: A study of variation. Am. J. Anat..

[48] Maeno T., Kobayashi K., Yamazaki N. (1997). Relationship between structure of finger tissue and location of tactile receptors. Trans. Jpn. Soc. Mech. Eng. C.

[49] Dai K., Wang X., Rojas A.M., Harber E., Tian Y., Paiva N., Gnehm J., Schindewolf E., Choset H., Webster-Wood V.A. Design of a biomimetic tactile sensor for material classification. Proceedings of the International Conference on Robotics and Automation.

[50] Yamada D., Maeno T., Yamada Y. Artificial finger skin having ridges and distributed tactile sensors used for grasp force control. Proceedings of the IEEE/RSJ International Conference on Intelligent Robots and Systems.

[51] Wandersman E., Candelier R., Debrégeas G., Prevost A. (2011). Texture-induced modulations of friction force: The fingerprint effect. Phys. Rev. Lett..

[52] Gerling G.J., Thomas G.W. The effect of fingertip microstructures on tactile edge perception. Proceedings of the Joint Eurohaptics Conference and Symposium on Haptic Interfaces for Virtual Environment and Teleoperator Systems, World Haptics Conference.

[53] Gerling G.J. (2010). SA-I mechanoreceptor position in fingertip skin may impact sensitivity to edge stimuli. Appl. Bionics Biomech..

[54] Yum S.M., Baek I.K., Hong D., Kim J., Jung K., Kim S., Eom K., Jang J., Kim S., Sattorov M. (2020). Fingerprint ridges allow primates to regulate grip. Proc. Natl. Acad. Sci. USA.

[55] Fagiani R., Massi F., Chatelet E., Berthier Y., Akay A. (2011). Tactile perception by friction induced vibrations. Tribol. Int..

[56] Maeno T., Kobayashi K. FE analysis of the dynamic characteristics of the human finger pad in contact with objects with/without surface roughness. Proceedings of the Dynamic Systems and Control.

[57] Scheibert J., Leurent S., Prevost A., Debrégeas G. (2009). The role of fingerprints in the coding of tactile information probed with a biomimetic sensor. Science.

[58] Adams M.J., Johnson S.A., Lefèvre P., Lévesque V., Hayward V., André T., Thonnard J.L. (2013). Finger pad friction and its role in grip and touch. J. R. Soc. Interface.

[59] Dzidek B.M., Adams M.J., Andrews J.W., Zhang Z., Johnson S.A. (2017). Contact mechanics of the human finger pad under compressive loads. J. R. Soc. Interface.

[60] Park G.S. (2023). The function of fingerprints: How can we grip?. Open Access Gov..

[61] Okamoto S., Konyo M., Tadokoro S. (2012). Discriminability-based evaluation of transmission capability of tactile transmission systems. Virtual Real..

[62] Hollins M., Bensmaïa S.J., Washburn S. (2001). Vibrotactile adaptation impairs discrimination of fine, but not coarse, textures. Somatosens. Mot. Res..

[63] Fox R.R., Maikala R.V., Bao S., Dempsey P.G., Brogmus G., Cort J., Maikala R.V. (2017). The relevance of psychophysical methods research for the practitioner. Proc. Hum. Factors Ergon. Soc. Annu. Meet..

[64] Han S.H., Song M., Kwahk J. (1999). A systematic method for analyzing magnitude estimation data. Int. J. Ind. Ergon..

[65] Lawrence M.A., Kitada R., Klatzky R.L., Lederman S.J. (2007). Haptic roughness perception of linear gratings via bare finger or rigid probe. Perception.

[66] Lederman S.J., Taylor M.M. (1972). Fingertip force, surface geometry, and the perception of roughness by active touch. Percept. Psychophys..

[67] Tanaka Y., Bergmann Tiest W.M., Kappers A.M.L., Sano A. (2014). Contact force and scanning velocity during active roughness perception. PLoS ONE.

[68] Wold S., Ruhe A., Wold H., Dunn W. (1984). The Collinearity Problem in Linear Regression. The Partial Least Squares (PLS) Approach to Generalized Inverses. Siam J. Sci. Stat. Comput..

[69] Manne R. (1987). Analysis of two partial-least-squares algorithms for multivariate calibration. Chemom. Intell. Lab. Syst..

[70] Wold S., Sjöström M., Eriksson L. (2001). PLS-regression: A basic tool of chemometrics. Chemom. Intell. Lab. Syst..

[71] Bastien P., Vinzi V.E., Tenenhaus M. (2005). PLS generalised linear regression. Comput. Stat. Data Anal..

[72] Stott A.E., Kanna S., Mandic D.P. (2018). Widely linear complex partial least squares for latent subspace regression. Signal Process..

[73] de Jong S. (1993). SIMPLS: An alternative approach to partial least squares regression. Chemom. Intell. Lab. Syst..

[74] MathWorks (2024). Plsregress: Partial Least-Squares (PLS) Regression.

[75] Walker S.G. (2021). A New Measure of Overlap: An Alternative to the *p*–value. arXiv.

[76] Eidous O., Daradkeh S. (2024). On inference of weitzman overlapping coefficient *Δ*(X,Y) in the case of two normal distributions. Int. J. Theor. Appl. Math..

[77] Yoshioka T., Gibb B., Dorsch A., Hsiao S.S., Johnson K.O. (2001). Neural coding mechanisms underlying perceived roughness of finely textured surfaces. J. Neurosci..

[78] Drewing K. (2018). Judged Roughness as a Function of Groove Frequency and Groove Width in 3D-Printed Gratings.

[79] Sathian K., Goodwin A.W., John K.T., Darian-Smith I. (1989). Perceived roughness of a grating: Correlationn with responses of mechanoreceptive afferents innervating the monkey’s fingerpad. J. Neurosci..

[80] Taylor M.M., Lederman S.J. (1975). Tactile roughness of grooved surfaces: A model and the effect of friction. Percept. Psychophys..

[81] Lederman S.J. (1974). Tactile roughness of grooved surfaces: The touching process and effects of macro-and microsurface structure. Percept. Psychophys..

[82] Kuroki S., Sawayama M., Nishida S. (2021). The roles of lower- and higher-order surface statistics in tactile texture perception. J. Neurophysiol..

